# Whey protein isolate supplementation improves body composition, muscle strength, and treatment tolerance in malnourished advanced cancer patients undergoing chemotherapy

**DOI:** 10.1002/cam4.2517

**Published:** 2019-09-30

**Authors:** Emanuele Cereda, Annalisa Turri, Catherine Klersy, Silvia Cappello, Alessandra Ferrari, Andrea Riccardo Filippi, Silvia Brugnatelli, Marilisa Caraccia, Silvia Chiellino, Valeria Borioli, Teresa Monaco, Giulia Maria Stella, Luca Arcaini, Marco Benazzo, Giuseppina Grugnetti, Paolo Pedrazzoli, Riccardo Caccialanza

**Affiliations:** ^1^ Clinical Nutrition and Dietetics Unit Fondazione IRCCS Policlinico San Matteo Pavia Italy; ^2^ Biometry and Clinical Epidemiology Service Fondazione IRCCS Policlinico San Matteo Pavia Italy; ^3^ Medical Oncology Unit Fondazione IRCCS Policlinico San Matteo Pavia Italy; ^4^ Radiation Oncology Unit Fondazione IRCCS Policlinico San Matteo Pavia Italy; ^5^ Unit of Respiratory System Diseases Fondazione IRCCS Policlinico San Matteo Pavia Italy; ^6^ Division of Hematology Fondazione IRCCS Policlinico San Matteo Pavia Italy; ^7^ Department of Molecular Medicine University of Pavia Pavia Italy; ^8^ Department of Otolaryngology University of Pavia Pavia Italy; ^9^ Head Neck Surgery Fondazione IRCCS Policlinico San Matteo Pavia Italy; ^10^ Nursing Technical and Rehabilitation Service Fondazione IRCCS Policlinico San Matteo Pavia Italy; ^11^ Department of Internal Medicine University of Pavia Pavia Italy

**Keywords:** cancer, malnutrition, nutritional counseling, whey proteins

## Abstract

In recent years, whey proteins (WP) have attracted increasing attention in health and disease for their bioactive functions. The aim of this study was to evaluate the benefit of WP isolate (WPI) supplementation in addition to nutritional counseling in malnourished advanced cancer patients undergoing chemotherapy (CT). In a single‐center, randomized, pragmatic, and parallel‐group controlled trial (http://www.ClinicalTrials.gov: NCT02065726), 166 malnourished advanced cancer patients with mixed tumor entities candidate to or undergoing CT were randomly assigned to receive nutritional counseling with (N = 82) or without (N = 84) WPI supplementation (20 g/d) for 3 months. The primary endpoint was the change in phase angle (PhA). Secondary endpoints included changes in standardized PhA (SPA), fat‐free mass index (FFMI), body weight, muscle strength, and CT toxicity (CTCAE 4.0 events). In patients with the primary endpoint assessed (modified intention‐to‐treat population), counseling plus WPI (N = 66) resulted in improved PhA compared to nutritional counseling alone (N = 69): mean difference, 0.48° (95% CI, 0.05 to 0.90) (*P* = .027). WPI supplementation also resulted in improved SPA (*P* = .021), FFMI (*P* = .041), body weight (*P* = .023), muscle strength (*P* < .001), and in a reduced risk of CT toxicity (risk difference, −9.8% [95% CI, −16.9 to −2.6]; *P* = .009), particularly of severe (grade ≥ 3) events (risk difference, −30.4% [95% CI, −44.4 to −16.5]; *P* = .001). In malnourished advanced cancer patients undergoing CT, receiving nutritional counseling, a 3‐month supplementation with WPI resulted in improved body composition, muscle strength, body weight, and reduced CT toxicity. Further trials, aimed at verifying the efficacy of this nutritional intervention on mid‐ and long‐term primary clinical endpoints in newly diagnosed specific cancer types, are warranted.

## Graphical abstract

Whey proteins (WP) have attracted increasing attention in health and disease for their bioactive functions. In malnourished advanced cancer patients undergoing chemotherapy (CT) and receiving nutritional counseling, a 3‐month supplementation with WP resulted in improved body composition, muscle strength, and reduced CT toxicity.

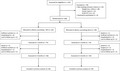

## 1 INTRODUCTION

Malnutrition is a frequent comorbidity in cancer patients, particularly in advanced disease requiring multidisciplinary interventions.^1^, ^2^, ^3^, ^4^

Nutritional problems such as weight loss (WL), low body mass index (BMI), and reduced protein‐calorie intake before and/or during chemotherapy (CT) and radiotherapy (RT) have been associated with worse prognosis, impaired quality of life (QoL), greater treatment toxicity, and severe mucositis.^3^, ^4^

Early nutritional intervention with high protein‐calorie intake can result in better nutritional status and QoL as well as in improved treatment tolerance.^5^, ^6^, ^7^, ^8^ Accordingly, nutritional counseling has been recommended for every cancer patient at nutritional risk, undergoing CT, and/or RT.^3^, ^4^

In recent years, whey protein (WP) supplementation has attracted increasing attention in health and disease.^9^

WP represents the soluble class of dairy proteins which makeup approximately 20% of the total bovine milk proteins,^10^ and are known immune‐enhancing constituents linked to a range of bioactive functions, such as prebiotic effects, promotion of tissue repair, maintenance of intestinal integrity, destruction of pathogens, and elimination of toxins.^11^, ^12^ WPs are rich in substrates for glutathione (GSH) synthesis^13^, ^14^ and the fact that cysteine is the limiting amino acid for the production of intracellular GSH is what makes them such an interesting potential dietary adjunct for cancer patients. Through its associated enzymes, GSH has a major role in cell protection against free radicals, ionizing radiation, reactive oxygen species, and carcinogens.^15^ Supplementation with WP may also induce more muscle protein synthesis than other protein sources, due to their faster digestion, leading to a more rapid increase in plasma amino acid levels, particularly in essential amino acids.^16^ Intervention studies have shown that WP supplementation improves muscle mass and function among sarcopenic older adults,^17^, ^18^ and preserves muscle mass during intentional WL in obese older adults.^19^ This is beneficial for cancer patients, considering their imbalance in protein metabolism,^20^ and the association between reduced muscle mass and increased CT toxicity.^21^

To date, the efficacy of WP isolate (WPI) supplementation in cancer patients has been investigated only in a few unpowered randomized trials, but an improvement in nutritional status and immunity parameters, QoL, functional status, and muscle strength, as well as in increased survival has reported.^22^, ^23^, ^24^

A major issue in daily practice is the availability of accurate and noninvasive technologies to evaluate and monitor the efficacy of nutritional interventions. The use of measured bioelectrical parameter, such as phase angle (PhA), using bioelectrical impedance vector analysis (BIVA), was demonstrated to reliably reflect cell integrity^25^ and energy balance in patients with chronic diseases.^26^, ^27^ In addition, BIVA appears to be an accurate procedure to evaluate the body composition, since it is independent of the hydration status.^25^ Several studies have shown that PhA and standardized PhA (SPA) can predict prognosis in different cancer populations^28^ and that PhA is a reliable marker for the detection of sarcopenia in cancer patients.^29^, ^30^

The aim of this randomized trial was to evaluate the effect of WPI supplementation on PhA and other predefined outcomes (SPA, body weight, muscle strength, fat‐free mass index [FFMI], QoL, and CT toxicity), over 3 months in malnourished advanced cancer patients undergoing CT, and receiving nutritional counseling as a standard of care.

## 2 METHODS

### 2.1 Study design

We performed a single‐center, randomized (1:1), pragmatic, and parallel‐group controlled clinical trial (NCT02065726; February 2014–June 2018). Allocation of the two intervention groups was performed according to a computer‐generated random blocks randomization list (varying block sizes). The randomization list was prepared by a local statistician, who was not involved in the selection and enrollment of patients. Concealment was achieved using sealed envelopes.

### 2.2 Participants

Adult (age ≥18 years), malnourished (6‐month unintentional WL ≥10%) advanced cancer patients (lung, stomach, esophagus, pancreas, colon, blood, breast, and head‐neck) candidate to or undergoing CT, were screened and considered eligible for study inclusion when they presented an Eastern Cooperative Oncology Group (ECOG) performance status ≤2^31^ and were not receiving any type of artificial nutrition (enteral or parenteral).

### 2.3 Interventions

In addition to standard CT regimens, patients were randomized to receive nutritional counseling with or without WPI supplementation, for 3 months. Nutritional counseling consisted of a personalized dietary prescription to achieve estimated protein‐calorie requirements. Total daily energy requirements were calculated by multiplying the estimated resting energy expenditure (Harris‐Benedict equation) by a correcting factor of 1.5. Daily protein requirements were set at 1.5 g/kg of actual body weight.^4^ Counseling included sample meal plans and recipe suggestions and it was tailored on personal eating patterns and food preferences, taking into account the nutrition impact symptoms (chewing difficulties, dysphagia, anorexia, dysgeusia, nausea, vomiting, diarrhea, and constipation). Additionally, it comprised the use of oral nutritional supplements (ONS; 1‐2 cans of energy‐dense, high protein oral formula, without any functional or immunomodulatory compound, providing approximately 300‐600 kilocalories and 20‐40 g of protein), which were prescribed when patients were unable to maintain satisfactory spontaneous food intake (<60% of estimated requirements for two consecutive weeks). Regular consultations with a registered dietician were also included, either monthly, by means of face‐to‐face interviews at the time of the scheduled follow‐up visits, or weekly by means of telephone interviews.^3^, ^8^, ^32^, ^33^, ^34^

If oral intakes were <60% of estimated requirements for two consecutive weeks despite the use of ONS, artificial nutrition support (enteral or parenteral as appropriate) was started.^3^, ^4^

In addition to nutritional counseling, patients allocated to the WPI group received two sachets/day of lipid‐ and lactose‐free, highly purified (microfiltration), and low temperature‐dried (cysteine‐rich) cow milk WP (Prother®; Difass International srl) providing 20 g of proteins. The WPI supplement was mixed both in water and in foods according to patients’ preferences. A short booklet of recipes with specific suggestions was also provided, in order to facilitate assumption. Caregivers and dietitians assessed and monitored compliance, recording with a diary the number of sachets consumed every day.

### 2.4 Evaluations

Information was collected based on age, gender, tumor localization, stage, and related treatments.

Body weight and height were measured using the same calibrated scale (to the nearest 0.1 kg; Wunder Sa.bi. Srl.) using a stadiometer and BMI was calculated.^35^ History of unintentional WL in the previous 6 months was obtained retrospectively. Phase angle, SPA, and FFMI were assessed by BIVA (NutriLAB, Akern/RJL) according to standard procedures^26^, ^27^ and muscle strength was measured by digital hand dynamometry (handgrip strength [HG]; DynEx™, Akern/MD Systems).

For the assessment of protein‐calorie intake at a baseline and throughout the study, participants were asked to provide detailed information on quality and quantities of food (including brand names of commercial and ready‐to‐eat‐foods, method of preparation, use of dressings, or added fat) and beverages consumed in the days before each visit (3‐day quantitative food diary + 24‐hour dietary recall) and telephone call (24‐h dietary recall). A validated atlas of food portions was also consulted to improve the accuracy of estimation.^8^, ^34^, ^36^, ^37^

Global QoL was assessed using the European Organization for the Research and Treatment of Cancer Quality of Life Questionnaire (EORTC QLQ‐C30).^38^

Finally, tolerance to CT was continuously monitored. In particular, patients were regularly examined by the same oncologists (blinded to treatment allocation) to assess the occurrence of toxicity according to the National Cancer Institute Common Toxicology Criteria (CTCAE 4.0).^39^

### 2.5 Outcomes

The primary outcome was the change in PhA at 3 months. Secondary outcome variables included: changes in PhA at 1 month; changes in SPA, FFMI, body weight, and HG at both 1 month and 3 months; changes in global QoL at 3 months; CT toxicity (any event; multiple events; Grade ≥3 events; Grade 5 events; events requiring the complete suspension of CT).

### 2.6 Adverse events associated with nutritional intervention

No serious adverse event associated with WPI supplementation was expected. Patients were actively monitored for the occurrence of gastrointestinal disorders (common adverse events).

### 2.7 Statistical analysis

In the absence of preliminary data, we estimated that to detect a clinically meaningful difference (mean treatment difference/standard deviation [effect size] = 0.5) for the primary outcome measure with a power of 80% and two‐tailed type I error <5%, at least 64 patients per arm reaching the primary endpoint evaluation were required.

The analysis was conducted following a modified intention‐to‐treat (ITT) principle. The change in PhA at 3 months was investigated in the set of patients reaching the primary endpoint evaluation (primary efficacy population) using a generalized linear regression model. Then a series of supportive analyses of the primary endpoint was performed. First, to perform full ITT analysis, we conducted chained multiple imputation of the missing primary endpoint, assuming that missing values were at random, since dropouts were not clinically and statistically different from patients remaining in the study (data not shown). Independent variables for the imputation were age, gender, tumor site and stage, BMI, WL, HG, protein‐calorie intake, and global QoL. The analysis of the primary endpoint was repeated in the imputed cohort, while accounting for multiple imputations. Second, we refitted the model of primary efficacy analysis on the cohort of patients receiving at least three CT cycles during the study, and on the subset of the supplemented patients consuming at least 50% of the study product (against the entire control group); we also fitted a model adjusting for baseline PhA, gender, and stage.

The effect of WP on changes in continuous secondary endpoints was analyzed in the primary efficacy population using a generalized linear regression model. Finally, the data on CT toxicity (endpoints on a binomial scale) were evaluated in the randomized population and in the subgroup of patients receiving at least three CT cycles, using the Fisher's exact test.

All patients consuming at least one sachet of WP were included in the safety analysis. The following causes of dropout were observed: death, lost to follow‐up, hospitalization, artificial nutrition, and withdrawal.

Descriptive statistics of continuous variables were reported as a mean and standard deviation or median, while categorical variables were presented as counts and percentages.

The study statistician was blinded to treatment assignment. All statistical analyses were performed using the STATA 15.1 statistical software (Stata Corporation). The level of significance was set at the two‐tailed *P*‐value <.05.

### 2.8 Ethics

The study was conducted in accordance with the Declaration of Helsinki and approved by the local Ethics Committee. All patients provided a written informed consent before the study entry.

## 3 RESULTS

**Figure 1:**
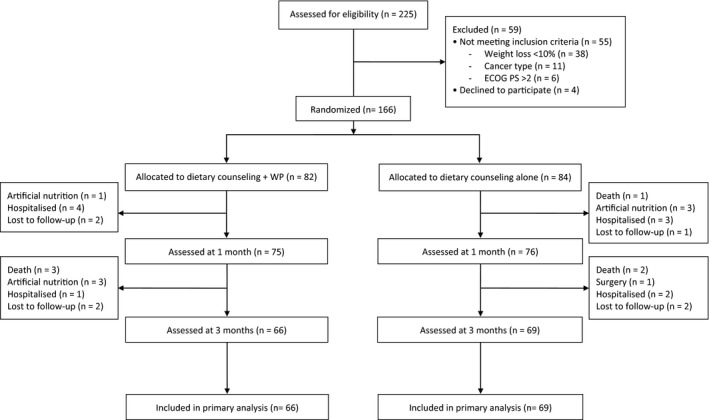
Study flow diagram

In total, 225 patients were screened for inclusion and 166 were randomized to interventions (Figure 1). Baseline characteristics in the two groups were comparable (Table 1). The enrollment was stopped when at least 64 patients per treatment arm had PhA (primary outcome variable) assessed at 3 months. Thirty‐one patients were lost before the assessment of the primary efficacy evaluation (15 in the first month, before the secondary efficacy evaluations). Hospitalizations were unrelated to nutritional interventions and were due to CT‐related toxicity (N = 6) or the worsening of clinical conditions (N = 4). The same was true for artificial nutrition (enteral nutrition, two cases; parenteral nutrition, five cases), which were the consequence of treatment‐related toxicity (N = 2) or the worsening of clinical conditions (N = 5). Six patients died, but all these events were unrelated to study interventions. The frequencies of different causes of dropout were similar for the two groups.

**Table 1:** Descriptive statistics of baseline characteristics according to the randomization group

Characteristic	Counseling (N = 84)	Counseling + whey protein (N = 82)
Male, N (%)	53 [63.1]	47 [57.3]
Age, mean (SD), y	65.7 (11.4)	65.1 (11.7)
≥65 y, N [%]	49 [58.3]	47 [57.3]
Diagnosis, N [%]		
Lung	20 [23.8]	23 [28.0]
Gastroesophageal	16 [19.0]	11 [13.4]
Pancreas	18 [21.4]	15 [18.3]
Colon	14 [16.7]	15 [18.3]
Blood	8 [9.5]	10 [12.2]
Breast	4 [4.8]	3 [3.6]
Head‐neck	4 [4.8]	5 [6.1]
Stage IV, N [%]	73 [86.9]	62 [75.6]
First‐line chemotherapy, N [%]	78 [92.9]	73 [89.0]
ECOG performance status, N [%]		
0	39 [46.4]	34 [41.5]
1	43 [51.2]	47 [47.3]
2	2 [2.4]	1 [1.2]
Body weight, mean (SD), kg	62.7 (13.2)	61.1 (13.4)
Body mass index, mean (SD), kg/m^2^	22.3 (3.9)	22.0 (4.1)
Six‐month weight loss, mean (SD), kg	13.4 (5.2)	13.6 (4.6)
Phase angle, mean (SD), °	5.16 (1.41)	5.17 (1.47)
Standardized phase angle, mean (SD)	0.09 (1.95)	0.16 (2.04)
Fat‐free mass index, mean (SD), kg/m^2^	18.4 (2.8)	18.0 (2.6)
Energy requirements^a^, mean (SD), kcal/d	1971 (312)	1926 (309)
kcal kg^−1^ d^−1^	31.9 (3.9)	32.0 (3.2)
Protein requirements, mean (SD), g kg^−1^ d^−1^	1.2 (0.1)	1.2 (0.1)
Energy intake, mean (SD), kcal/d	1460 (353)	1484 (407)
kcal kg^−1^ d^−1^	24.0 (6.8)	25.0 (7.4)
% of requirements	75 (19)	78 (21)
Protein intake, mean (SD), g/d	57.1 (14.8)	59.9 (18.8)
g kg^−1^ d^−1^	0.94 (0.28)	1.02 (0.37)
Handgrip strength, mean (SD), kg	22.1 (8.8)	21.2 (8.1)
Global QoL^b^, mean (SD), score	58.7 (20.5)	61.0 (16.3)

Abbreviations: ECOG, Eastern Cooperative Oncology Group; QoL, quality of life; SD, standard deviation.

a Calculated using the Harris‐Benedict equation multiplied by a correction factor of 1.5.

b Assessed by means of the European Organization for the Research and Treatment of Cancer Quality of Life Questionnaire Core 30 (EORTC‐QLQ‐C30).

Patients received a mean of three CT cycles (counseling + WPI, 3.0 ± 1.2 [≥3, N = 54]; counseling alone, 3.0 ± 1.2 [≥3, N = 58]).

Both study interventions were effective in maintaining and improving calorie and protein intakes; 58 patients required the prescription of ONS (counseling + WP, N = 21; counseling alone, N = 37; for comparison, *P* = .015). Compliance to WPI supplementation was fair (mean ± SD, 1.2 ± 0.6 sachets/day; ≥50%, N = 43). Nonetheless, the consumption of the study product was sufficient to result in a significantly higher change in mean protein intake: +8.5 g/d [95% CI, 2.4 to 14.6] (*P* = .007) (+0.10 g kg^−1^ d^−1^ [95% CI, 0.01 to 0.20]; *P* = .049). Mean protein intakes (mean ± SD) in the two groups were: counseling + WPI, 1.26 ± 0.38 g kg^−1^ d^−1^; counseling alone, 1.05 ± 0.30 g kg^−1^ d^−1^. No difference between groups was observed in the change of calorie intake: adjusted mean difference, +0.40 kcal kg^−1^ d^−1^ [95% CI, −1.76 to 2.55]; *P* = .72 (mean intake during study: counseling + WPI, 29.1 ± 7.6 kcal kg^−1^ d^−1^; counseling alone, 26.7 ± 6.9 kcal kg^−1^ d^−1^).

### 3.1 Primary endpoint

In the primary efficacy population (modified ITT population), the mean change in PhA at 3 months in the WPI group was +0.20° (95% CI, −0.12 to 0.52) as compared to −0.28° (95% CI, −0.56 to 0.01) in the control group: mean difference, 0.48° (95% CI, 0.05 to 0.90) (*P* = .027; Table 2).

**Table 2:** Effect of supplementation with whey protein on body composition, body weight, muscle strength, and quality of life in the modified intention‐to‐treat population (changes from baseline values)

Endpoint	First follow‐up visit (1 mo)				End of study (3 mo)			
	Counseling (N = 76) Mean (SD)	Counseling + whey protein (N = 75) Mean (SD)	Treatment effect Mean (95% CI)	*P*‐value	Counseling (N = 69) Mean (SD)	Counseling + whey protein (N = 66) Mean (SD)	Treatment effect Mean (95% CI)	*P*‐value
Phase angle (°)	−0.22 (1.23)	0.20 (1.14)	0.42 (0.04 to 0.80)	**.031**	−0.28 (1.18)	0.20 (1.29)	0.48 (0.05 to 0.90)	**.027**
Standardized phase angle	−0.39 (1.70)	0.39 (1.59)	0.78 (0.25 to 1.31)	**.004**	−0.36 (1.55)	0.33 (1.86)	0.69 (0.11 to 1.27)	**.021**
Fat‐free mass index (kg/m^2^)	−0.01 (1.26)	0.11 (1.17)	0.12 (−0.27 to 0.51)	.53	−0.14 (1.35)	0.32 (1.22)	0.46 (0.02 to 0.90)	**.041**
Body weight (kg)	−0.1 (2.3)	0.3 (2.3)	0.4 (−0.3 to 1.2)	.22	−0.7 (4.2)	1.0 (4.1)	1.7 (0.2 to 3.1)	**.023**
Handgrip strength (kg)	−0.4 (2.9)	0.3 (2.8)	0.7 (−0.2 to 1.6)	.12	−0.9 (4.4)	1.4 (3.1)	2.3 (1.0 to 3.6)	**<.001**
Global QoL a (score)	—	—	—	—	0.54 (16.5)	2.94 (13.3)	2.40 (−2.71 to 7.51)	.35

*P*‐values <.05 have been highlighted in bold.

Abbreviations: 95% CI, 95% confidence interval; QoL, quality of life; SD, standard deviation.

a Assessed by means of the European Organization for the Research and Treatment of Cancer Quality of Life Questionnaire Core 30 (EORTC‐QLQ‐C30).

All sensitivity analyses yielded results consistent with the primary analysis. Multiple imputation of missing outcomes resulted in a mean difference between groups of +0.40° (95% CI, 0.02 to 0.79) (*P* = .040), while in patients receiving at least three CT cycles the adjusted mean difference was +0.48° (95% CI, 0.03 to 0.92) (*P* = .035). Finally, after excluding patients with low compliance (<50% of prescribed WPI dose), the treatment effect was computed to +0.64° (95% CI, 0.18 to 1.11) (*P* = .007). When adjusting for baseline PhA, gender, and stage, the treatment effect was computed to + 0.47° (95% CI, 0.12 to 0.82) (*P* = .027).

### 3.2 Secondary outcomes

A significant effect of WPI supplementation on PhA was already present at 1 month (Table 2). Changes in SPA at both time points were consistent with those in PhA. A significant difference in the change in FFMI (*P* = .041), body weight (*P* = .023), and HG (*P* < .001) in favor of WPI was also found at 3 months. No significant differences were found in the change of QoL (*P* = .35) at the end of the study. Finally, the effect of WPI on CT toxicity was investigated (Table 3). In the randomized population, the WPI group had a lower risk of toxicity than the group receiving counseling alone (*P* = .009), particularly of multiple toxicity (*P* = .007) and severe toxicity events (*P* = .001). The sensitivity analysis restricted to patients receiving at least three CT cycles, confirmed these findings.

**Table 3:** Treatment toxicity according to CTCAE 4.03 in the randomized population

Endpoints	Primary analysis				Sensitivity analysis^a^			
	Counseling (N = 84) n [%]	Counseling + whey protein (N = 82) n [%]	Risk difference % (95% CI)	*P* value	Counseling (N = 58) n [%]	Counseling + whey protein (N = 54) n [%]	Risk difference % (95% CI)	*P* value
Any	83 [98.8]	73 [89.0]	−9.8 (−16.9 to −2.6)	**.009**	57 [98.3]	48 [88.9]	−9.3 (−18.4 to −0.4)	.055
Hematological^b^	17 [20.2]	10 [12.2]	−8.0 (−19.3 to 3.2)	.21	14 [24.1]	4 [7.4]	−16.7 (−29.8 to −3.7)	**.020**
Gastrointestinalc	44 [52.4]	38 [46.3]	−6.0 (−21.2 to 9.2)	.44	29 [50.0]	24 [44.4]	−5.6 (−24.1 to 12.9)	.58
Others	64 [76.2]	57 [69.5]	−6.7 (−20.2 to 6.8)	.38	41 [70.7]	39 [72.2]	1.5 (−15.2 to 18.3	>.99
Multiple	41 [48.8]	23 [28.0]	−20.8 (−35.2 to −6.3)	**.007**	26 [44.8]	13 [24.1]	−20.7 (−37.9 to −3.6)	**.029**
Grade 3‐4	44 [52.4]	18 [22.0]	−30.4 (−44.4 to −16.5)	**.001**	33 [56.9]	10 [18.5]	−38.4 (−54.8 to −21.9)	**<.001**
Hematological^b^^,^ d	12 [14.3]	4 [4.9]	−9.4 (−18.4 to −0.4)	.06	10 [17.2]	1 [1.9]	−15.4 (−25.8 to −5.0)	**.009**
Gastrointestinal^c^^,^ d	21 [25.0]	10 [12.2]	−12.8 (−24.7 to −0.9)	**.046**	18 [31.0]	6 [11.1]	−19.9 (−34.5 to −5.4)	**.012**
Others^d^	11 [13.1]	4 [4.9]	−8.2 (−16.9 to 0.5)	.10	5 [8.6]	3 [5.6]	−3.1 (−12.6 to 6.5)	.72
Grade 5	2 [2.4]	1 [1.2]	−1.2 (−5.2 to 2.9)	>.99	1 [1.7]	0 [0]	−1.7 (−5.2 to 1.8)	>.99
Complete CT suspension	10 [11.9]	5 [6.1]	−5.8 (−14.5 to 2.9)	.28	3 [5.2]	2 [3.7]	−1.5 (−9.1 to 6.2)	>.99

*P*‐values <.05 have been highlighted in bold.

Abbreviations: 95% CI, 95% confidence interval; CT, chemotherapy.

a Patients receiving at least three CT cycles during the study.

b Neutropenia/anaemia/thrombocytopenia.

c Nausea/vomiting/diarrhea/constipation.

d Only the worst was counted for each patient.

In both analysis populations, risk reduction in Grade ≥3 events was mainly for gastrointestinal toxicity.

### 3.3 Adverse events

No apparent gastrointestinal intolerance event was recorded. As reported above, six patients died during the study, but no death was related to the study intervention. No other intervention‐related adverse events occurred.

## 4 DISCUSSION

In the present trial, we found that the additional provision of WPI to malnourished cancer patients receiving nutritional counseling during CT, improved body composition, muscle strength, body weight, and resulted in reduced CT toxicity.

This is the first adequately powered study supporting the efficacy of this particular nutritional intervention in combination with nutritional counseling, although there is some previous positive evidence supporting WPI supplementation in cancer patients.^22^, ^23^, ^24^ In advanced non–small cell lung cancer, WPI supplementation resulted in increased body weight, survival, HG, and some domains of QoL at 6 months.^22^ In colon cancer patients, a clinically meaningful improvement in functional walking capacity was achieved before surgery,^23^ while increased GSH levels and improved nutritional status and immunity parameters were observed in cancer patients undergoing CT.^24^ Our results are also consistent with those achieved with the administration of WP‐based oral nutritional supplements aimed at regaining muscle mass and function in sarcopenic older adults.^17^, ^18^

Individualized nutritional counseling, including the use of ONS, is currently recommended as the standard of care for all cancer patients at nutritional risk, receiving anti‐cancer treatments.^3^, ^4^ Guidelines recommend providing 25‐30 kcal kg^−1^ d^−1^ and up to 1.2‐1.5 g of protein/kg/d,^3^, ^4^ although satisfying estimated requirements in all patients may be difficult. In our trial, the mean calorie intake was above the lower limit of recommendations in both groups, while the mean protein intake was satisfactory only in the WPI group, suggesting the importance of this macronutrient in cancer patients. Indeed, WPI possesses many distinctive properties and potential applications due to its amino acid profile and native structure. WPs are rich in cysteine, an essential substrate for GSH synthesis, which could play a protective role in cells under the high oxidative stress conditions induced by CT.^13^, ^14^, ^15^ WPs also contain high levels of other essential amino acids, such as leucine, which makes them an important food source for sustaining muscle protein anabolism and function.^16^, ^17^, ^18^, ^19^ Protein breakdown is upregulated in cancer patients and results in muscle weakness and dysfunction.^20^ However, anabolic pathways and recovery may still be preserved if appropriate amounts of essential amino acids are provided.^40^

We can argue that in our trial all positive outcomes were linked with each other. A higher protein‐calorie intake resulted in improved body composition and muscle function, which is also related to muscle protein stores. Reduced muscle mass has been associated with dose‐limiting toxicity in patients receiving systemic therapy 21 and improved body composition in patients supplemented with WPI is likely to have contributed to increased treatment tolerance. Supplementation resulted in a lower incidence of multiple and severe toxicity events, particularly gastrointestinal, which are likely to have a negative impact on spontaneous food intake. This might explain why fewer patients in the WPI group required ONS prescription to increase protein‐calorie intake.

Therefore, our trial highlights the importance of integrating appropriate nutritional care in malnourished patients receiving anti‐cancer treatment, in order to interrupt a downward spiral, potentially resulting in worse clinical outcome.^3^, ^4^, ^8^, ^34^

We acknowledge that our study has some limitations. First, our findings apply only to malnourished patients with different cancer types, receiving CT for a short period. However, considering the high prevalence of malnutrition in oncology, we cannot exclude a potentially positive effect also in nonmalnourished patients, and in the medium to long term. This certainly requires further investigation by adequately designed trials, involving specific cancer‐type populations. Indeed, nutritional counseling is the standard of care and WPI supplementation should be considered only in combination with it. Second, this was a single‐site trial, which restricts data generalizability, though it facilitated a homogeneous approach to all patients.

Finally, it would have been interesting to explore the effect of WPI in a double‐blinded trial, comparing different protein sources. However, we chose a pragmatic study design, which closely resembles real‐world clinical practice.

In conclusion, in malnourished advanced cancer patients undergoing CT and receiving nutritional counseling, a 3‐month supplementation with WPI resulted in improved body composition, muscle strength, body weight, and reduced CT toxicity, which may lead to improved treatment efficacy. Further trials, aimed at verifying the efficacy of this nutritional intervention on mid‐ and long‐term primary clinical endpoints in newly diagnosed specific cancer types, are warranted.

## CONFLICT OF INTEREST

The authors have no conflict of interest to declare.

## AUTHOR CONTRIBUTIONS

Dr Caccialanza, Dr Cereda, Dr Klersy, and Dr Pedrazzoli had full access to all of the data in the study, and take responsibility for the integrity of the data and the accuracy of the data analysis. Riccardo Caccialanza, Emanuele Cereda, and Catherine Klersy conceptualized and designed the study. Riccardo Caccialanza, Annalisa Turri, and Alessandra Ferrari provided the administrative support. Luca Arcaini, Marco Benazzo, Valeria Borioli, Alessandra Ferrari, Riccardo Caccialanza, Silvia Cappello, Marilisa Caraccia, Emanuele Cereda, Andrea Riccardo Filippi, Silvia Brugnatelli, Teresa Monaco, Paolo Pedrazzoli, Silvia Chiellino, Giulia Maria Stella, and Annalisa Turri provided the study materials or patients. Luca Arcaini, Marco Benazzo, Valeria Borioli, Alessandra Ferrari, Riccardo Caccialanza, Silvia Cappello, Marilisa Caraccia, Emanuele Cereda, Andrea Riccardo Filippi, Catherine Klersy, Silvia Brugnatelli, Teresa Monaco, Paolo Pedrazzoli, Silvia Chiellino, Giulia Maria Stella, and Annalisa Turri collected and assembled the data. Riccardo Caccialanza, Emanuele Cereda, Catherine Klersy, Paolo Pedrazzoli, and Annalisa Turri analyzed and interpreted the data. Riccardo Caccialanza, Emanuele Cereda, and Catherine Klersy wrote the manuscript. All authors approved the final manuscript. All authors are accountable for all aspects of the work.

## Data Availability

The data that support the findings of this study are available from the corresponding author upon reasonable request.
